# Cutaneous nerve fiber pathology and function in Parkinson’s disease and atypical parkinsonism – a cohort study

**DOI:** 10.1038/s41531-025-01030-y

**Published:** 2025-06-15

**Authors:** Mattias Andréasson, Wojciech Paslawski, Astrid Juhl Terkelsen, Kristin Samuelsson, Henrik Zetterberg, Kaj Blennow, Páll Karlsson, Per Svenningsson

**Affiliations:** 1 Center for Neurology, Academic Specialist Center, Stockholm, Sweden; 2 Department of Neurology, Karolinska University Hospital, Stockholm, Sweden; 3 Department of Clinical Neuroscience, Karolinska Institutet, Stockholm, Sweden; 4 Department of Neurology, Aarhus University Hospital, Aarhus, Denmark; 5 Department of Psychiatry and Neurochemistry, Institute of Neuroscience and Physiology, the Sahlgrenska Academy at the University of Gothenburg, Mölndal, Sweden; 6 Clinical Neurochemistry Laboratory, Sahlgrenska University Hospital, Mölndal, Sweden; 7 Department of Neurodegenerative Disease, UCL Institute of Neurology, Queen Square, London, UK; 8 UK Dementia Research Institute at UCL, London, UK; 9 Hong Kong Center for Neurodegenerative Diseases, Hong Kong, China; 10 Wisconsin Alzheimer’s Disease Research Center, University of Wisconsin School of Medicine and Public Health, University of Wisconsin-Madison, Madison, WI USA; 11 Paris Brain Institute, ICM, Pitié-Salpêtrière Hospital, Sorbonne University, Paris, France; 12 Neurodegenerative Disorder Research Center, Division of Life Sciences and Medicine, and Department of Neurology, Institute on Aging and Brain Disorders, University of Science and Technology of China and First Affiliated Hospital of USTC, Hefei, China; 13 Danish Pain Research Center, Health, Aarhus University, Aarhus, Denmark

**Keywords:** Parkinson's disease, Parkinson's disease, Neurology, Movement disorders, Parkinson's disease

## Abstract

There is scientific evidence for ongoing neurodegeneration and alpha-synuclein pathology involving the peripheral nervous system in Parkinson’s disease (PD) and multiple system atrophy (MSA). We explored putative disease-mirroring properties of cutaneous nerve fibers in patients with PD (*n* = 20), MSA (*n* = 12), four-repeat tauopathies (*n* = 11), and controls (*n* = 20). Assessments included clinical rating scales, blood sampling, sudomotor testing, skin punch biopsies from the neck and leg, and 1-year follow-up. Skin alpha-synuclein seeding amplification assay (SAA) and determination of intraepidermal nerve fiber density (IENFD) were performed. Reduced electrochemical skin conductance was evident in MSA, associated with clinical rating scores. Cervical skin SAA (PD vs controls) achieved a 100% sensitivity and 70% specificity for detecting PD. We found no difference in baseline IENFD, nor in 1-year changes, in patients relative to controls. Baseline IENFD, plasma neurofilament light, and SAA kinetics associated with 1-year clinical disease progression in MSA. Skin may harbor promising prognostic properties in MSA.

## Introduction

Small fiber neuropathy has repeatedly been demonstrated in the context of Parkinson’s disease (PD), with involvement of both autonomic^1^ and somatosensory^2^ nerve fibers. Furthermore, cutaneous denervation has been suggested as a biomarker of disease progression in PD^3,4^. In light of studies detecting pathological alpha-synuclein (α-syn) in skin biopsies, either through immunohistochemistry^5^ or seeding amplification assays (SAA)^6^, neurodegeneration extending beyond the central nervous system in PD has been proposed. This may be of great importance as disease progression from peripheral to central, in the proposed *body first* subtypes^7^, may allow detection before central neurodegeneration.

Small fiber neuropathy and pathological α-syn deposits have been demonstrated in skin biopsies from patients with multiple system atrophy (MSA)^8,9^. In contrast, studies are scarce with respect to peripheral neurodegenerative changes in progressive supranuclear palsy (PSP) and corticobasal degeneration (CBD), the two other main disease entities within atypical parkinsonism. Atypical parkinsonism remains a challenging differential diagnosis in the evaluation of patients presenting with parkinsonism. Moreover, prognosis and symptomatic treatment options differ substantially from PD, why additional diagnostic and prognostic minimally invasive tools are needed.

With the present study, we aimed to evaluate cutaneous small fibers focusing on structural, functional, and pathological features, in a cohort of participants with PD, atypical parkinsonism, and healthy controls (HC). We sought to detect possible cross-sectional discriminative features and associations with measures of disease burden. Using a longitudinal study design, we further aimed to explore potential markers of disease progression over time.

## Results

Two controls were excluded after a review of medical records demonstrating historic exposure to a cytotoxic agent and a history of foot surgery with associated peripheral nerve damage, respectively. The final study population consisted of 63 participants who gave informed consent: PD (*n* = 20), HC (*n* = 20), MSA (*n* = 12), and four-repeat-tauopathies (4R-tauopathies) (*n* = 11).

### Clinical characteristics at baseline

Groups were comparable regarding age and sex, with a female predominance in all groups. Disease duration and age at onset differed significantly between groups. Participants with 4R-tauopathies and MSA exhibited a higher modified Hoehn & Yahr (mHY) stage relative to participants with PD (post-hoc pairwise comparison, *p* < 0.001). Plasma levels of neurofilament light (NfL) differed significantly between groups with post hoc pairwise comparisons indicating higher levels in participants with MSA and 4R-tauopathies (HC vs MSA, *p* < 0.001; HC vs 4R-tauopathies, *p* < 0.001; HC vs PD, *p* = 0.33; PD vs MSA, *p* = 0.064; PD vs 4R-tauopathies, *p* = 0.020; MSA vs 4R-tauopathies, *p* = 1.0). Baseline data are further detailed in Table 1.

**Table 1:** Clinical and demographic characteristics of the study cohort (*n* = 63)

	PD (*n* = 20)	MSA (*n* = 12)	4R (*n* = 11)	HC (*n* = 20)	*p*
		[MSA-P, *n* = 8; MSA-C, *n* = 4]	[PSP-RS, *n* = 7; CBD, *n* = 4]		
Age at examination (y), median (IQR)	63.2 (14.2)	59.3 (12.7)	68.6 (10.5)	62.9 (6.3)	0.084
Sex (M/F)	7/13	3/9	4/7	7/13	0.95
Smoking (*n*), (% yes)	1 (0.050)	1 (0.083)	0 (0)	1 (0.050)	1.0
Ethnicity (White/Asian/Hispanic/North African)	17/2/1/0	12/0/0/0	9/1/0/1	20/0/0/0	0.22
Disease characteristics					
Onset age (y), median (IQR)	57.6 (13.6)	52.0 (11.1)	65.0 (12.5)	.	**0.041**
Disease duration (y), median (IQR)	6.5 (4.9)	5.8 (1.6)	4.4 (3.1)	.	**0.046**
L-dopa duration (y), median (IQR)	4.1 (4.4)	2.7 (4.3)	0.2 (2.6)	.	**0.024**
LEDD (mg), median (IQR)	593 (549)	688 (1201)	200 (600)	.	**0.049**
mHY (stage), median (IQR)	2.0 (0)	4.0 (2.0)	4.0 (2.0)	.	**<0.001**
MDS-UPDRS part III (*p*), median (IQR)	23 (15)	.	.	.	.
UMSARS part II (*p*), median (IQR)	.	29.5 (18)	.	.	.
UMSARS part IV (*p*), median (IQR)	.	3 (1)	.	.	.
PSP-CDS (*p*), median (IQR)	.	.	11 (9)	.	.
SCOPA-AUT (*p*), median (IQR)	10.5 (10)	31.5 (20)	13 (20)	5.5 (7)	**<0.001**
Max. systolic BP fall (mmHg), median (IQR)	8.5 (7)	16 (18)	5 (20)	9.5 (15)	0.34
Max. diastolic BP fall (mmHg), median (IQR)	0.0 (8)	8.5 (10)	1.0 (12)	0.5 (11)	**0.033**
UENS *(p*), median (IQR)	5.0 (5)	3.0 (3)	3.0 (4)	2.0 (2)	**0.004**
p-neurofilament light (pg/mL), median (IQR)	14.4 (8.9)	32.4 (23.7)	28 (29.1)	9.8 (6.2)	**<0.001**

Overview of the study cohort. Data are presented with non-parametric descriptive statistics. Group comparisons performed with Fisher’s exact test and Kruskal–Wallis H-test.

*PD* Parkinson’s disease, *MSA-P/C* multiple system atrophy with predominant parkinsonism/cerebellar features, *4R* four-repeat tauopathy, *CBD* corticobasal degeneration, *PSP-RS* probable progressive supranuclear palsy–Richardson syndrome, *HC* healthy control, *IQR* interquartile range, *LEDD* L-dopa equivalent daily dose, *mHY* modified Hoehn & Yahr, *MDS-UPDRS* Movement Disorders Society Unified Parkinson’s Disease Rating Scale, *UMSARS* Unified Multiple System Atrophy Rating Scale, *PSP-CDS* Progressive Supranuclear Palsy Clinical Deficits Scale, *SCOPA-AUT* Scales for Outcomes in Parkinson’s Disease-Autonomic Dysfunction, *BP* blood pressure, *UENS* Utah Early Neuropathy Scale, *p* plasma.

Bold indicates *p* < 0.05.

### Baseline autonomic assessments reveal reduced electrochemical skin conductance in MSA

Blood pressure (BP) measurements at active stand test revealed significant group differences for the maximum fall in diastolic, but not systolic, BP. Post hoc pair-wise comparisons demonstrated a significantly higher diastolic BP fall in participants with MSA compared with PD (*p* = 0.034). The Scales for Outcomes in Parkinson’s disease-Autonomic Dysfunction (SCOPA-AUT) differed significantly between groups, with higher scores in participants with MSA (Table 1).

**Fig. 1:**
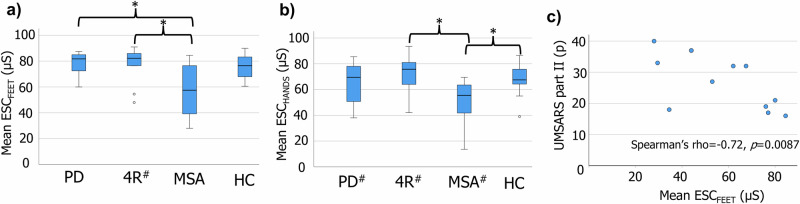
Baseline mean skin conductance and associations with disease burden in MSA. Mean ESC measured from feet (**a**) and hands (**b**). **c** Significant association between UMSARS part II and ESC_FEET_ in participants with MSA. *p < 0.05. PD Parkinson’s disease, 4R four-repeat tauopathies, MSA multiple system atrophy, HC healthy controls, UMSARS Unified Multiple System Atrophy Rating Scale, ESC electrochemical skin conductance, µS microsiemens, hash symbol one missing value.

Figure 1a, b shows mean electrochemical skin conductance (ESC) values recorded from feet and hands. Significant group differences were seen at both locations (ESC_FEET_: *H* = 12.2, *p* = 0.007; ESC_HANDS_ *H* = 11.0, *p* = 0.012). Post hoc pairwise comparisons suggested these differences were driven by a lower ESC in participants with MSA (ESC_FEET_: MSA vs PD, *p* = 0.010; MSA vs HC, *p* = 0.12; MSA vs 4R-tauopathies, *p* = 0.019; PD vs HC, *p* = 1.0; PD vs 4R-tauopathies, *p* = 1.0; HC vs 4R-tauopathies, *p* = 1.0; ESC_HANDS_: MSA vs PD, *p* = 0.054; MSA vs HC, *p* = 0.033; MSA vs 4R-tauopathies, *p* = 0.018; PD vs HC, *p* = 1.0; PD vs 4R-tauopathies, *p* = 1.0; HC vs 4R-tauopathies, *p* = 1.0). A subgroup analysis in participants with MSA showed significant negative associations between ESC_FEET_ and the Unified Multiple System Atrophy Rating Scale part II (rho = −0.72, *p* = 0.0087, Fig. 1c) and mHY (rho = −0.61, *p* = 0.034). No significant associations were seen for UMSARS part IV (*p* = 0.059) or SCOPA-AUT (*p* = 0.85). Nor were any associations observed with regard to ESC_HANDS_, or in other disease groups.

### Cutaneous small fiber density comparable between groups at baseline

**Fig. 2:**
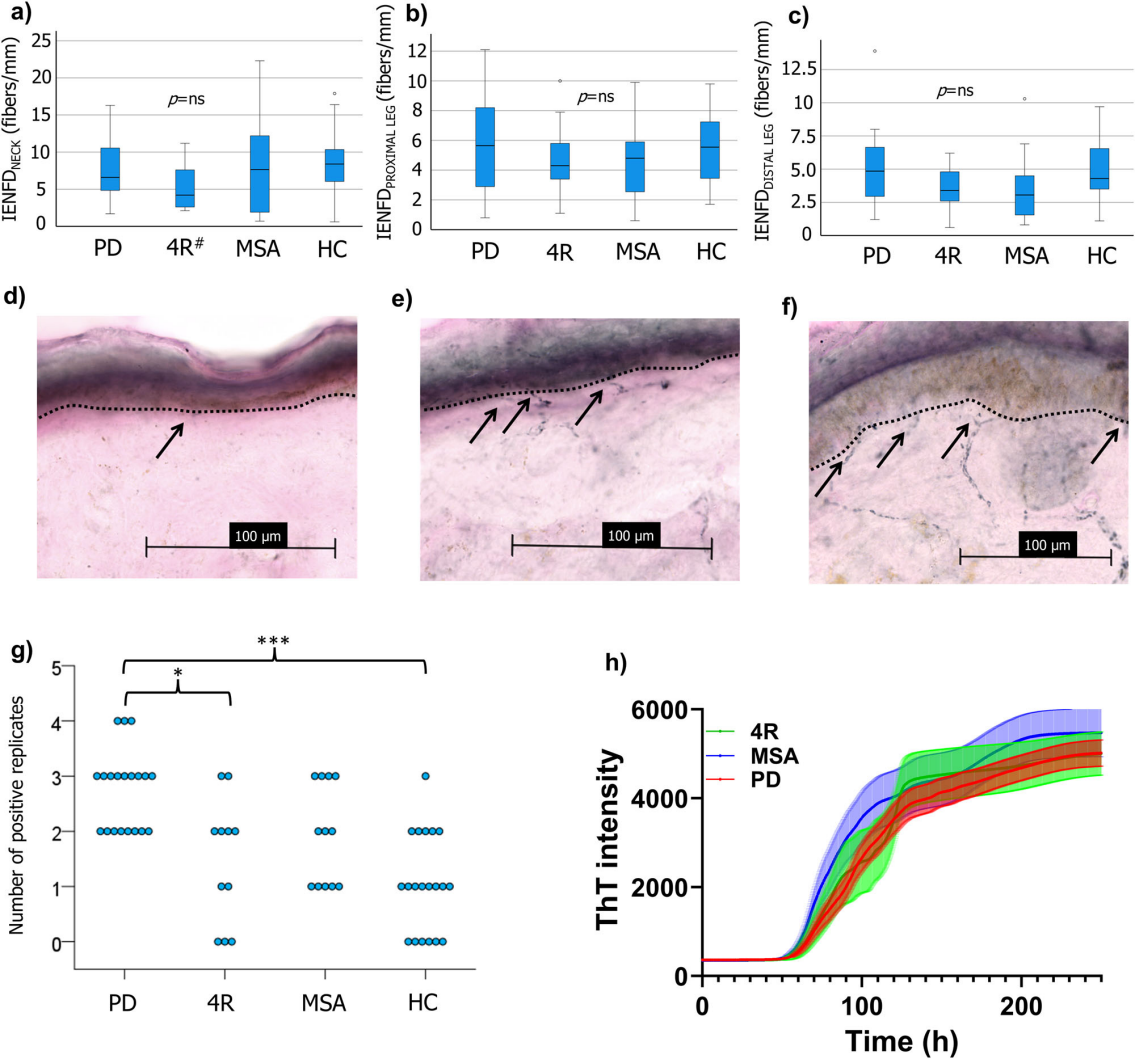
Baseline intraepidermal nerve fiber density and cervical skin seeding amplification assay. Comparable IENFD between groups at the neck (**a**), proximal (**b**), and distal leg (**c**). Sagittal skin sections from participants with PD (**d**), MSA (**e**), and controls (**f**). Arrows show fibers traversing to the epidermis. **g** Number of positive replicates per participant from cervical skin SAA. **h** Kinetic SAA properties in patient samples, based on mean values from all positive replicates. *p < 0.05, ***p < 0.001. IENFD intraepidermal nerve fiber density, PD Parkinson’s disease, 4R four-repeat tauopathies, MSA multiple system atrophy, HC healthy control, ThT thioflavin T, hash symbol one missing value.

No significant group differences in intraepidermal nerve fiber density (IENFD) were observed at the neck (*H* = 2.8, *p* = 0.42), proximal leg (*H* = 1.6, *p* = 0.67), or distal leg (*H* = 5.2, *p* = 0.15) (Fig. 2a–c). No significant cross-sectional associations were seen between IENFD and clinical rating scores (PD: the International Parkinson and Movement Disorder Society sponsored revision of the Unified Parkinson’s Disease Rating Scale (MDS-UPDRS) part III, *p* > 0.5; 4R-tauopathies: the Progressive Supranuclear Palsy Clinical Deficits Scale (PSP-CDS), *p* > 0.3; MSA: UMSARS part II and IV, *p* > 0.3). Sweat glands were visualized in biopsies from the distal leg in a subset of participants, enabling the quantification of sweat gland nerve fiber length density (SGNFLD) (PD, *n* = 13; 4R-tauopathies, *n* = 10; MSA, *n* = 7; HC, *n* = 15). No significant group differences (*H* = 1.4, *p* = 0.71) or cross-sectional associations with measures of disease burden were observed.

### Patterns of cervical α-syn seeding activity at baseline

The number of positive cervical SAA replicates differed significantly between groups (*H* = 24.8, *p* < 0.001). Post hoc comparisons demonstrated significant differences between participants with PD and controls (*p* < 0.001), and 4R-tauopathies (*p* = 0.015), favoring a higher positivity rate in participants with PD. The number of positive replicates for each participant and SAA kinetics based on positive replicates from patients are illustrated in Fig. 2g, h. Applying a post hoc threshold of ≥2 positive replicates for a positive test, 20/20 PD, 6/11 4R-tauopathies, 7/12 MSA patients, and 6/20 controls were positive. The sensitivity and specificity for detecting a synucleinopathy (MSA or PD) were 84.4% (exact 95% CI: 67.2, 94.7) and 61.3% (exact 95% CI: 42.2, 78.2), respectively. Comparing only participants with PD with controls demonstrated a sensitivity of 100% (exact 95% CI: 83.2, 100) and specificity of 70% (exact 95% CI: 45.7, 88.1) for the detection of PD. No associations between kinetic variables, mean maximum fluorescence signal (mean max. signal), and mean lag phase time, and baseline clinical measures were observed.

One participant with a 4R-tauopathy declined a biopsy taken from the neck, instead proximal leg was used for the SAA. The same was done for one control at follow-up.

### Participants in the one-year follow-up

56 participants returned for the 1-year follow-up. Drop-outs (*n* = 7) were attributed to death (*n* = 2) and worsened cognitive and/or physical status precluding participation (*n* = 5). Re-evaluation of clinical diagnosis in patients did not render any change from baseline. Thus, longitudinal results are based on: PD (*n* = 19), MSA (*n* = 8), 4R-tauopathies (*n* = 9), and HC (*n* = 20). The median time between baseline and 1-year visits was 1.01 years, and no significant differences between groups (*p* = 0.49).

### One-year changes in IENFD, SGNFLD and skin conductance

The median ΔIENFD did not differ between groups at the neck (*H* = 0.56, *p* = 0.91 [PD: 1.6 fibers/mm, IQR 5.0 fibers/mm; MSA: 0.85 fibers/mm, IQR 2.2 fibers/mm; 4R-tauopathies: 2.0 fibers/mm, IQR 2.6 fibers/mm; HC: 0.9 fibers/mm, IQR 2.7 fibers/mm]), proximal leg (*H* = 3.7, *p* = 0.30 [PD: 0.70 fibers/mm, IQR 2.3 fibers/mm; MSA: 1.8 fibers/mm, IQR 2.4 fibers/mm; 4R-tauopathies: −0.10 fibers/mm, IQR 2.4 fibers/mm; HC: 1.1 fibers/mm, IQR 2.9 fibers/mm]) or distal leg (*H* = 2.4, *p* = 0.49 [PD: 0.5 fibers/mm, IQR 1.6 fibers/mm; MSA: 0.95 fibers/mm, IQR 2.6 fibers/mm; 4R-tauopathies: −0.10 fibers/mm, IQR 2.0 fibers/mm; HC: 0.10 fibers/mm, IQR 4.2 fibers/mm]). Due to limited longitudinal SGNFLD data in participants with MSA and 4R-tauopathies, median ΔSGNFLD was calculated only for participants with PD and controls (*n* = 12 and 13, respectively). ΔSGNFLD did not differ between participants with PD and controls (Mann–Whitney U test *z* = −0.44, *p* = 0.69 [PD: −19.5 mm^−2^, IQR 372 mm^−2^; HC: −63 mm^−2^, IQR 324 mm^−2^]).

The median change in ESC_HANDS_ did not differ between groups (*H* = 3.4, *p* = 0.34 [PD: −2.5 µS, IQR 14.8 µS; MSA: −3.0 µS, IQR 10.4 µS; 4R-tauopathies: −12 µS, IQR 29.0 µS; HC: 0.0 µS, IQR 11.4 µS]). For ΔESC_FEET_, a significant group difference was demonstrated (*H* = 7.8, *p* = 0.049 [PD: −0.50 µS, IQR 4.3 µS; MSA: −1.5 µS, IQR 18.3 µS; 4R-tauopathies: −11.5 µS, IQR 17.5 µS; HC: −1.5 µS, IQR 11.9 µS]). Post hoc comparisons showed a significantly larger reduction in skin conductance in participants with 4R-tauopathies relative to PD (*p* = 0.034), but not controls (*p* = 0.61).

### Associations between baseline variables and longitudinal changes in clinical rating scores

In participants with MSA, baseline IENFD associated with ΔUMSARS part II (neck: rho = −0.93, *p* < 0.001; proximal leg: rho = −0.71, *p* = 0.047; distal leg: rho = −0.88, p = 0.0039), part IV (neck: rho = −0.76, *p* = 0.030; distal leg: rho = −0.78, *p* = 0.022) and ΔmHY (distal leg: rho = −0.76, *p* = 0.027). Moreover, baseline SAA kinetic parameters showed similar associations with ΔUMSARS part II (mean lag phase time: rho = −0.88, *p* = 0.0039; mean max. signal: rho = 0.83, *p* = 0.010), part IV (mean lag phase time: rho = −0.78, *p* = 0.022; mean max. signal: rho = 0.85, *p* = 0.0079) and ΔmHY (mean lag phase time: rho = −0.76, *p* = 0.027; mean max. signal: rho = 0.87, *p* = 0.0047). All correlation analyses, including positive associations with plasma neurofilament light, are shown in Table 2. Plots for the significant associations are found in the Supplementary Data File 1.

**Table 2:** Associations between baseline variables and 1-year changes in clinical rating scores in MSA

	IENFD_NECK_ (fibers/mm)	IENFD_PROXIMAL LEG_ (fibers/mm)	IENFD_DISTAL LEG_ (fibers/mm)	p-NfL (pg/mL)	Mean lag phase time (h)	Mean max. signal (a.u.)	ESC_FEET_ (µS)	ESC_HANDS_ (µS)
ΔUMSARS part II (*p*)	−0.93***	−0.71*	−0.88**	0.76*	−0.88**	0.83*	ns	ns
ΔUMSARS part IV (*p*)	−0.76*	ns	−0.78*	0.78*	−0.78*	0.85**	ns	ns
ΔmHY (stage)	ns	ns	−0.76*	0.87**	−0.76*	0.87**	ns	ns

Correlation analyses between baseline IENFD, plasma NfL, skin conductance, and kinetic SAA variables, and delta changes in clinical rating scores were carried out in MSA (n = 8). Significant correlation coefficients (Spearman’s rho) unadjusted for multiple comparisons are presented, *p < 0.05; **p < 0.01; ***p < 0.001. After Bonferroni adjustment for multiple comparisons (eight comparisons within each outcome measure) associations remained significant between: ΔUMSARS part II and IENFD_NECK_ (*p* < 0.01), IENFD_DISTAL LEG_ (*p* < 0.05), mean lag phase time (*p* < 0.05); ΔmHY and NfL (*p* < 0.05), mean max. signal (*p* < 0.05).

*IENFD* intraepidermal nerve fiber density, *p-NfL* plasma neurofilament light, *ESC* electrochemical skin conductance, *SAA* seeding amplification assay, *ns* not significant, Δ*UMSARS* 1-year change in the Unified Multiple System Atrophy Rating Scale, Δ*mHY* 1-year change in the modified Hoehn and Yahr scale.

## Discussion

In the present study we explored structural and functional characteristics of cutaneous small nerve fibers in controls and in a cohort of patients with PD, MSA, and 4R-tauopathies. First, we found no discriminative features for baseline IENFD (Fig. 2a–c) at any anatomical site, or SGNFLD at the distal leg, and no evidence of significant 1-year progression of cutaneous denervation in patients relative to controls. Second, assessment of sudomotor function, as measured by Sudoscan^®^, suggested sudomotor dysfunction associated with baseline measures of disease burden in participants with MSA. Third, cervical skin SAA showed promising results for the sensitivity in detecting PD, with all participants with PD showing two or more positive replicates (Fig. 2g). Lastly, putative cutaneous baseline markers associated with 1-year changes in clinical disease progression were suggested in participants with MSA.

The proposed underlying rationale for the measurement of ESC entails the electrical stimulation of postganglionic sudomotor nerve fibers and/or their end organ (eccrine sweat glands). The methodology has been suggested to reflect the integrity of postganglionic sudomotor fibers^10^. Our ESC results in participants with PD are consistent with two previous studies^11,12^ showing no difference relative to controls. However, a reduced ESC was evident in the present study for participants with MSA, with pairwise comparisons demonstrating lower values relative to participants with PD and 4R-tauopathies in feet, and controls and 4R-tauopathies in hands (Fig. 1a, b). Furthermore, measurements from feet associated cross-sectionally with mHY and UMSARS part II in participants with MSA (Fig. 1c). Similar findings have been reported when comparing MSA to PD^13^ or controls^14,15^. Whether the findings in MSA reflect an underlying selective degeneration of postganglionic fibers or a mere nerve dysfunction is unclear. Since IENFD only reflects somatosensory nerve fibers, further analysis of SGNFLD could give further insight. Indeed, postganglionic involvement in MSA has been suggested previously by means of quantitative sudomotor axon reflex testing^16^ and quantification of SGNFLD^9^. In the present study, no significant group differences at baseline were demonstrated for SGFNLD, possibly supporting an underlying postganglionic nerve dysfunction explaining our results. However, we acknowledge that SGNFLD data were only available for seven participants with MSA at baseline, and ESC was measured from the sole of the foot while SGNFLD was determined from biopsies collected 10 cm proximally to the lateral malleolus.

Our study did not detect any differences in baseline IENFD between the groups. In relation to PD, previous studies have reported contradictory findings. Some have shown a significant reduction in IENFD in participants with PD compared to controls, both in early, treatment naïve^1^ and later stages^2,5,17^. However, others reported no such difference in mean IENFD^18^. Similarly, conflicting results exist for MSA, with both comparable^17^ and reduced^9^ IENFD reported relative to controls. A possible explanation for our findings in PD may relate to the increasingly appreciated hypothesis that disease manifestations in PD reflect different origins and spreading paths of the underlying α-syn pathology, the so-called *brain-first vs body-first* hypothesis^7^. In other words, a high proportion of participants with *brain-first* PD could be reflected by less disease involvement of the peripheral nervous system. The comparable orthostatic BP reactions, partly dependent on peripheral autonomic fibers, in participants with PD and controls may support this explanation (Table 1).

Participants with PD demonstrated a higher SAA positivity rate relative to controls and 4R-tauopathies. The calculated sensitivity for the detection of PD is comparable to previous studies in skin^19–21^, although with a lower specificity in the present study. For the detection of synucleinopathies (PD + MSA), both sensitivity and specificity were lower. Possible explanations include the presence of co-pathologies, such as Lewy bodies, in the older participants with 4R-tauopathies. A previous autopsy study reported the presence of Lewy-related pathology in 8% of PSP cases^22^. Furthermore, although not clinically apparent during longitudinal follow-up, the possibility of incidental Lewy body pathology in controls remains and could be reflected by the SAA positivity in some controls.

Few studies have assessed longitudinal trajectories of IENFD in PD. We show here that IENFD does not progressively decrease over a 1-year period in participants with PD, MSA, or 4R-tauopathies relative to controls. Previous studies assessing longitudinal changes in IEFND have suggested positive findings in PD^3,4,23^, however, in these studies, controls were only assessed at baseline. We believe the combined analysis of patients and controls, at both baseline and follow-up, strengthens our results, arguing against significant cutaneous denervation over a 1-year period in participants with PD.

Longitudinal evaluations allowed for the assessment of 1-year clinical disease progression by calculating Δ values from clinical rating scores. Although we could not detect baseline factors associated with 1-year clinical progression in participants with PD and 4R-tauopathies, exploratory findings were evident in MSA. A lower IENFD at baseline, at all three biopsy locations, associated with a higher degree of clinical 1-year progression in participants with MSA (Table 2). Given that no reduction of IEFND was demonstrated in participants with MSA over the same period, we speculate that clinical worsening may deteriorate faster than cutaneous denervation. Although a small sample size, we believe these data are novel and may motivate larger studies in early MSA to further delineate whether IENFD at baseline may carry prognostic properties.

Similar associations could not be demonstrated in participants with PD and 4R-tauopathies. Given the slower rate of clinical progression in PD and the availability of symptomatic treatment, we believe a longer follow-up is needed to discern whether baseline IENFD may be associated with longitudinal disease progression in PD.

The 1-year follow-up did not demonstrate any significant reduction in ESC values in participants with MSA relative to controls. However, given the cross-sectional associations in participants with MSA, we believe further studies are needed to determine if reduced ESC is also prevalent in early MSA and whether the methodology may harbor prognostic properties. Regarding participants with PD, we cannot exclude that a longer follow-up time might have shed light on possible predictive properties for the measurement of skin conductance. In this regard, it should be noted that a reduction in ESC has been reported in PD patients with REM Sleep Behavior Disorder (RBD) relative to both controls and PD patients without RBD^24^. Moreover, a high prevalence of abnormal skin wrinkle test, suggestive of autonomic small fiber dysfunction, has been suggested in PD^25^. Thus, a longer follow-up time could reveal whether reduced ESC at baseline may serve as a marker of a more aggressive *body-first* PD phenotype.

We found exploratory associations between kinetic parameters (mean lag phase time and mean max. signal) and 1-year changes in clinical rating scores in participants with MSA (Table 2). Interestingly, SAA kinetics have previously been suggested to differ between MSA and PD when performed in cerebrospinal fluid^26^. Although such differences were not demonstrated in this study, we believe our results motivate further evaluation of SAA kinetics as a prognostic marker in MSA.

The main study limitations pertain to small sample size, in particular with regard to participants with MSA and 4R-tauopathies. This limitation is primarily related to the rarity and severity of these diseases. The use of a multicenter approach could have mitigated this limitation. Given the smaller number of participants with MSA, all correlation analyses were considered exploratory and should be interpreted with caution.

A diagnosis of polyneuropathy formed part of our exclusion criteria. Thus, we cannot exclude that some participants with PD and polyneuropathy, where PD may have been a contributing etiology, were excluded from the study, possibly decreasing the proportion of participants with *body first* PD. On the other hand, PD participants with undiagnosed polyneuropathy may also have been included in the study, however, considering that we did not demonstrate significant group differences in IENFD we believe this limitation not to be substantial. Moreover, the inclusion of controls with subclinical polyneuropathy cannot be excluded and may have clouded possible group differences. However, a screening of controls for subclinical conditions would have questioned the generalizability of our findings.

Two participants with PD were under treatment with amitriptyline, in which 24 h drug withdrawal may not have been sufficient time to exclude remaining pharmacodynamic effects. This limitation would not have affected the ESC results found in the participants with MSA.

We believe the longitudinal design conferred strengths to the study, including the high retention of participants with atypical parkinsonism at the 1-year follow-up. The follow-up visit furthermore allowed for the possible reclassification and consolidation of clinical diagnosis, and analysis of factors associating with clinical progression over time, as we report in MSA.

The IENFD delta values were, for the most part of what we could expect, as there will always be some variations between immunolabeling performed in different batches. The findings indicate stable IENFD values between the two time points, although a potential batch effect (in either direction) cannot be excluded. Importantly, however, since controls were included at both time points, the absence of significant changes relative to controls still substantiate our interpretation of results. A longer time frame for the follow-up visit might have been more appropriate in order to capture divergent denervation patterns in participants with PD relative to controls, where 1 year may have been too short to demonstrate significant differences. Given the more rapid disease course in atypical parkinsonism, the 1-year follow-up was chosen to ensure a high retention of participants with MSA and 4R-tauopathies.

We acknowledge that the genetic status of the participants with PD was not evaluated. It has been shown that monogenic PD is not always associated with underlying α-syn pathology (e.g., as seen in *LRRK2*-PD and *PRKN*-PD)^27^, and thus possibly influences SAA results. However, given the rarity of pathogenic *LRRK2* variants in Sweden^28^ and the absence of any PD participant with motor onset before the age of 40, we would expect a low prevalence of these variants in our cohort.

Given the strict exclusion criteria, we acknowledge that our findings may not be fully representative for the general disease population.

In conclusion, a progressive reduction of IENFD appears not evident in participants with PD or atypical parkinsonism, relative to controls over a 1-year time frame. Although patients and controls had comparable IEFND at baseline, possible prognostic properties of baseline IENFD may be evident in participants with MSA. Low ESC is prevalent in participants with MSA and may indicate postganglionic disease involvement. Skin SAA is a promising tool for the detection of PD, although with limited specificity in this cohort. Further studies are warranted to shed light on whether baseline kinetic SAA properties, together with IENFD, may carry prognostic value in MSA.

## Methods

### Participants and study design

This was a longitudinal study with assessments performed at baseline and at a 1-year follow-up. Participants were recruited between April 2021 and November 2022. Inclusion was carried out during outpatient visits to Center for Neurology in Stockholm. Spouses and accompanying persons were asked to participate as sex- and age-matched controls. All participants gave informed consent. The study protocol was approved by the Swedish Ethical Review Authority (ref. nr 2020-04341) and the research was performed in accordance with the Declaration of Helsinki.

The inclusion criteria:

- Diagnosis of probable PD^29^, probable MSA^30^, probable PSP^31^ or possible CBD^32^ according to established criteria.
- Age 40–80 years.

The exclusion criteria:

- Medical record stating a diagnosis of diabetes mellitus, polyneuropathy, systemic lupus erythematosus, rheumatoid arthritis, Sjogren’s syndrome, or systemic vasculitis.
- Ongoing drug abuse or heavy alcohol consumption^33^.
- Historic exposure to agents associated with peripheral neuropathy.
- Ongoing infection with HIV.
- Unable to withdraw peroral drugs conferring a main anticholinergic effect, including tricyclic antidepressants, for 24 h.

The criteria were identical for patients and controls except for parkinsonism. Given their pathological and phenotypic overlap, patients with PSP and CBD were categorized into a single group entitled 4R-tauopathies.

### Clinical assessments

Demographic and clinical information were obtained through oral interviews. Disease duration was defined as the time from motor onset until the study inclusion date. Disease burden was assessed in participants with clinical rating scales: MDS-UPDRS^34^ part III in PD, UMSARS^35^ part II + IV in MSA, and PSP-CDS^36^ in PSP and CBD. The mHY^37,38^ stage was applied to all patients. Levodopa equivalent daily dose (LEDD) was calculated according to the published conversion formula^39^. Clinical signs of peripheral neuropathy were evaluated with the Utah Early Neuropathy Scale^40^.

### Blood biochemistry

Fasting venous blood samples were collected in the morning. To exclude known causes of peripheral neuropathy, levels of homocysteine, methylmalonic acid, folate, cobalamin, glucose, glycated hemoglobin, liver enzymes, thyroid-stimulating hormone, creatinine, electrolytes and HIV serology were measured. NfL concentration in EDTA plasma samples, stored at −80 °C and shipped on dry ice to the Clinical Neurochemistry Laboratory, Sahlgrenska University Hospital/Mölndal, was measured using Single molecule array technology on an HD-X analyzer according to instructions from the kit manufacturer (Quanterix, Billerica, MA).

### Autonomic assessments

BP was measured with an automatic sphygmomanometer in the supine position after a 5-min rest, followed by repeated measurements in the standing position after 0, 1, and 3 min. SCOPA-AUT^41^ was applied in all participants. ESC was assessed using the Sudoscan^®^. Mean values from feet and hands were recorded. If conductance was only measurable from one body side, this was reported as the mean value.

### Skin biopsy

Under local anesthesia, four skin punch biopsies (3 mm) were collected from each participant: C8 cervical level (*n* = 2 in each participant), 20 cm distal to the anterior iliac spine (*n* = 1 in each participant), and 10 cm proximal to the lateral malleolus (*n* = 1 in each participant). In patients, the body side most affected by motor deficits was chosen, given previous reports of asymmetry regarding small fiber neuropathy in PD^1,42^. Quantification of IENFD was performed at all three biopsy sites. In the distal leg, SGNFLD was also determined. The fourth biopsy, from the neck, was used for SAA.

### Quantification of IENFD and SGNFLD

Biopsies were directly placed in tubes containing Zamboni’s fixative for 16–22 h. After washing, biopsies were stored in tubes filled with cryoprotectant and shipped to Aarhus, Denmark, for further analysis and quantification of IENFD following published guidelines^43^. A detailed description of the method can be found elsewhere^44^. Briefly, three 50-μm-cryostat sections (Microm Cryostat M 500 OM, Zeiss, Germany) were immunostained with rabbit anti-human PGP 9.5 (1:1000; Zytomed Systems, Berlin, Germany) and appropriate secondary antibody to visualize the nerve fibers (1:200; Vector Laboratories, Burlingame, CA). The number of nerve fibers entering the epidermis was then quantified by dividing the fiber count by the length of the skin section, with all assessments conducted in a blinded manner to estimate IENFD.

SGNFLD, the total length density of nerve fibers innervating the sweat glands, was assessed using global spatial sampling, an unbiased stereological counting method, and the NewCAST software (Visiopharm, Hoersholm, DK). Global spatial sampling is described in detail elsewhere^45,46^. Briefly, isotropic virtual planes with random orientation within a virtual 3D box are superimposed over the sweat glands. This ensures that every fiber within the sweat gland has an equal probability of being quantified, as all fibers that intersect a nerve fiber are selected. The total length of the fibers within the volume of the sweat glands is estimated using a mathematical equation described in detail elsewhere^45,46^.

### Skin tissues and preparation for cervical SAA

Skin tissues were weighed, and approximately 10 mg each were washed 3 times in 1× Tris(hydroxymethyl)aminomethane–buffered saline. The skin homogenates were prepared in 10% (weight/volume) lysis buffer containing 2 mmol of calcium chloride and 0.25% (weight/volume) collagenase A (Roche) in Tris-buffered saline and incubated at 37 °C for 4 h, followed by sonication. The tissue debris was removed by centrifuging for 5 min at 5000 × *g*, and the supernatant fraction was collected.

### Preparation of recombinant α-syn monomers

α-syn monomers were prepared as described previously^47^, with minor modifications. Briefly, pET11-D vector, containing the insert coding human α-syn, was expressed in *E.coli* BL21(DE3) competent cells using an auto-induction method. Cells were harvested by centrifugation and treated with the osmotic shock buffer (20 mM Tris-HCl, pH 7.2, 40% sucrose), incubated for 10 min, and centrifuged again. Afterwards, the pellet was suspended in ice-cold deionised water, with subsequent addition of saturated MgCl_2_, and briefly incubated on ice. The periplasmic fraction of the cell lysate was collected and the majority of unwanted proteins precipitated by adjusting pH to 3.5 with 1 M HCl. Soluble proteins were collected by centrifugation and the pH of the obtained supernatant was adjusted to pH 7.5 with 1 M NaOH. The solution was filtered and fractionated on a Q-Sepharose column connected to an ÄKTA Go system (GE Healthcare) using a rising concentration of NaCl. Fractions containing α-syn were identified by SDS-PAGE and pulled together. Further, high molecular weight aggregates were removed by filtration through a 30 kDa cut-off filter to obtain only the monomeric form of αSN and analyzed with SDS-PAGE to ensure purity. Next, to remove endotoxins, the solution was incubated with Lipid Removal Agent (Merck) according to manufacturer's instructions and finally dialyzed against deionised water. The α-syn concentration was determined using BCA assay (Thermo Scientific), protein was aliquoted, lyophilized, and stored at −20 °C.

### Seeding amplification assay

SAA was performed as previously described with minor modifications^6^. The SAA reaction mix was composed of 40 mmol of phosphate buffer (pH 8.0), 170 mmol of sodium chloride, 0.1 mg/mL of recombinant human wild-type α-syn, 10 μmol of thioflavin T (ThT), and 0.00125% sodium dodecyl sulfate. One 3/32″ Si3N4 bead and 98-μL per well of the reaction mix was added per well into a black 96-well plate with a clear bottom and seeded with 2 μL of skin homogenate. The plate was placed in the FLUOstar Omega plate reader, set to 37 °C and the reaction started. Each plate was shaken for 1 min every 29 min, and fluorescence was measured at 485 nm after each cycle. The plate was continuously shaken/read for ~150 h. Four replicates were performed for each participant sample. The following two kinetic variables were determined as a mean of all replicates: mean lag phase time, with negative replicates set to 125 h, and mean maximum fluorescence signal (mean max. signal). A positive replicate was defined as having a lag phase <125 h and a maximal fluorescence signal more than background fluorescence plus ten standard deviations (~1000 units in our setup)^48^.

### One-year follow-up

All participants were invited to participate in a 1-year follow-up where all assessments were repeated.

### Statistical analysis

Numerical and ordinal variables were tested for normality using the Shapiro-Wilk test and assessment of skewness. Most data demonstrated a non-normal distribution and therefore analyses were performed with non-parametric testing. In cross-sectional data, numerical and ordinal data are presented as median (interquartile range) and group comparisons were done using the Kruskal–Wallis *H*-test. In significant findings, post-hoc Dunn’s test with Bonferroni adjustment were performed for pairwise comparisons. Categorical variables are presented as proportions, and group comparisons were performed using chi-square or Fisher’s test where appropriate. Correlation analyses were performed with Spearman’s rank correlation test. A power analysis conducted a priori (mean difference in ESC of 10 µS, SD 12 µS) showed that 23 participants per group were necessary to demonstrate a significant difference between two groups with 80% power.

For the longitudinal part, only participants with observations at baseline and follow-up were included in the statistical analysis. Delta (Δ) values were calculated for clinical rating scores, IENFD, and ESC by subtraction (i.e., Δ*A* = *A*_one-year follow-up_ – *A*_baseline_). Correlation analyses between changes in clinical rating scores and baseline variables were performed using Spearman’s rank correlation test. A two-tailed *p*-value < 0.05 was considered significant. All analyses were performed using IBM SPSS Statistics, version 27.0.1.0.

## Supplementary information

SUPPLEMENTARY FILE

## Data Availability

Anonymized data not published within this article will be shared upon request from any qualified investigator.
