# Supplementary material for: Cutaneous nerve fiber pathology and function in Parkinson’s disease and atypical parkinsonism – a cohort study
Source: NPJ Parkinsons Dis. 2025 Jun 15;11:170. doi: 10.1038/s41531-025-01030-y (PMC12167382; doi:10.1038/s41531-025-01030-y)
Supplement: Supplementary file 1 — SUPPLEMENTARY FILE [file 41531_2025_1030_MOESM1_ESM.pdf]

SUPPLEMENTARY FILE 1.

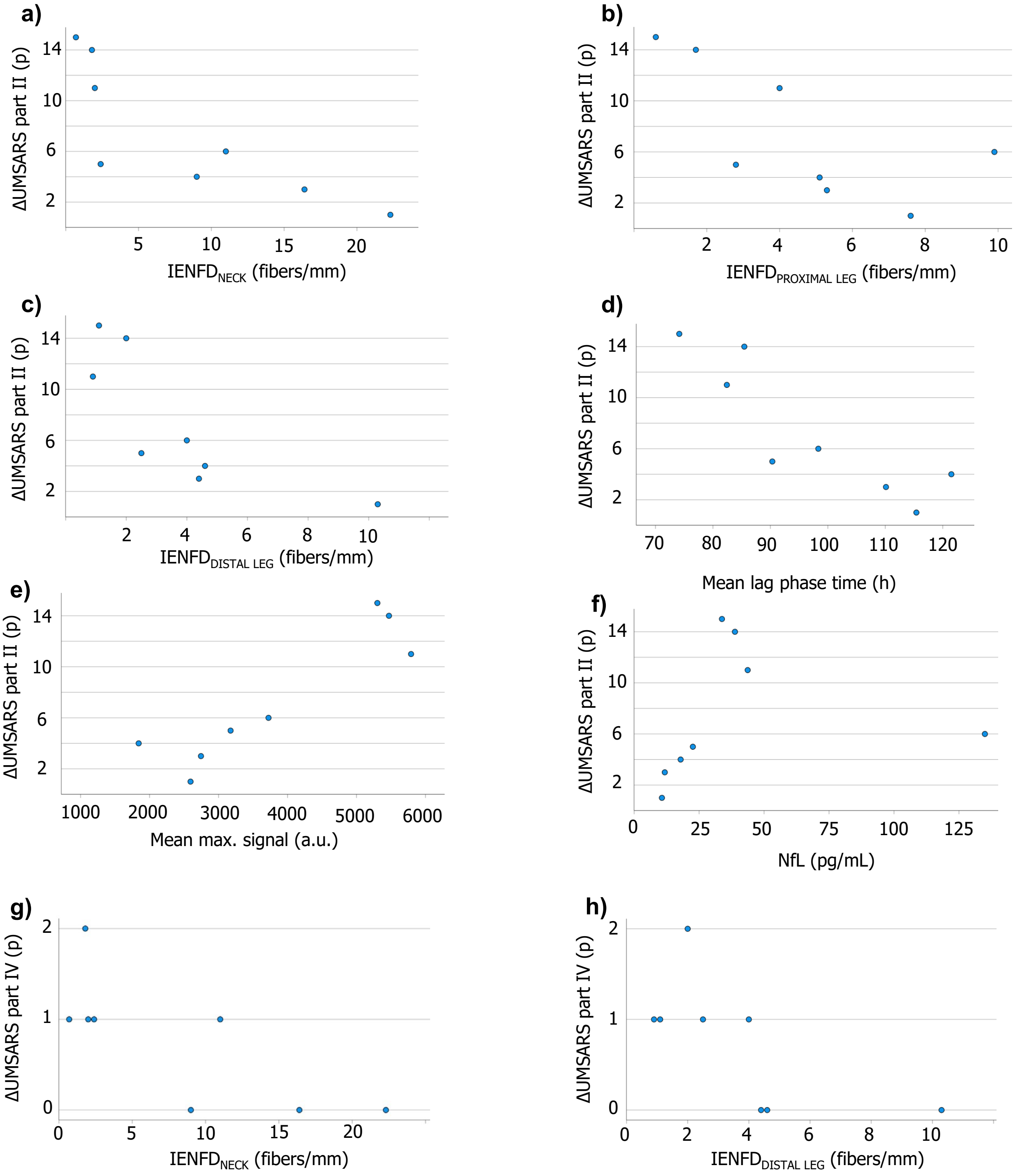

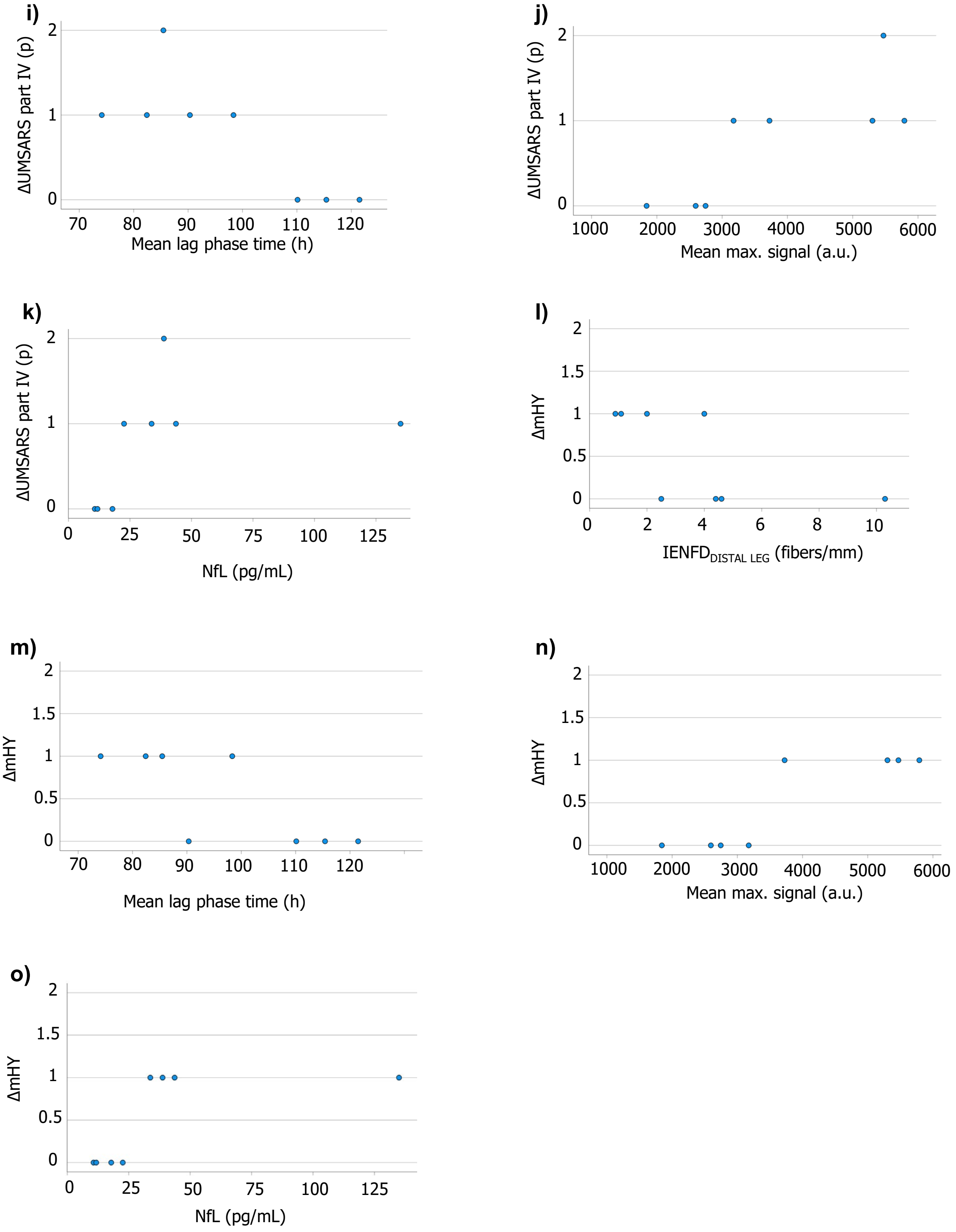

i)
ΔUMSARS part IV (p)
Mean lag phase time (h)
j)
ΔUMSARS part IV (p)
Mean max. signal (a.u.)
k)
ΔUMSARS part IV (p)
NfL (pg/mL)
l)
ΔmHY
IENFD DISTAL LEG (fibers/mm)
m)
ΔmHY
Mean lag phase time (h)
n)
ΔmHY
Mean max. signal (a.u.)
o)
ΔmHY
NfL (pg/mL)

Supplementary fig. 1 Scatter plots illustrating associations between baseline markers and longitudinal clinical disease progression in MSA (n=8).

Clinical disease progression was measured by obtaining one-year delta values for the clinical rating scales UMSARS part II **(a-f)**, UMSARS part IV **(g-k)** and mHY **(l-o)**. The shown scatter plots correspond to the significant Spearman's correlation coefficients presented in Table 2.

Abbreviations: IENFD – intraepidermal nerve fiber density; NfL – plasma neurofilament light; ESC – electrochemical skin conductance; ΔUMSARS – 1-year change in the Unified Multiple System Atrophy Rating Scale; ΔmHY – 1-year change in the modified Hoehn and Yahr scale
